# A Computational Framework to Benchmark Basket Catheter Guided Ablation in Atrial Fibrillation

**DOI:** 10.3389/fphys.2018.01251

**Published:** 2018-09-21

**Authors:** Martino Alessandrini, Maddalena Valinoti, Laura Unger, Tobias Oesterlein, Olaf Dössel, Cristiana Corsi, Axel Loewe, Stefano Severi

**Affiliations:** ^1^Department of Electronic Engineering and Information Technology, University of Bologna, Cesena, Italy; ^2^Institute of Biomedical Engineering, Karlsruhe Institute of Technology, Karlsruhe, Germany

**Keywords:** atrial fibrillation, ablation, basket catheter, computational modeling, rotor

## Abstract

Catheter ablation is a curative therapeutic approach for atrial fibrillation (AF). Ablation of rotational sources based on basket catheter measurements has been proposed as a promising approach in patients with persistent AF to complement pulmonary vein isolation. However, clinically reported success rates are equivocal calling for a mechanistic investigation under controlled conditions. We present a computational framework to benchmark ablation strategies considering the whole cycle from excitation propagation to electrogram acquisition and processing to virtual therapy. Fibrillation was induced in a patient-specific 3D volumetric model of the left atrium, which was homogeneously remodeled to sustain reentry. The resulting extracellular potential field was sampled using models of grid catheters as well as realistically deformed basket catheters considering the specific atrial anatomy. The virtual electrograms were processed to compute phase singularity density maps to target rotor tips with up to three circular ablations. Stable rotors were successfully induced in different regions of the homogeneously remodeled atrium showing that rotors are not constrained to unique anatomical structures or locations. Density maps of rotor tip trajectories correctly identified and located the rotors (deviation < 10 mm) based on catheter recordings only for sufficient resolution (inter-electrode distance ≤3 mm) and proximity to the wall (≤10 mm). Targeting rotor sites with ablation did not stop reentries in the homogeneously remodeled atria independent from lesion size (1–7 mm radius), from linearly connecting lesions with anatomical obstacles, and from the number of rotors targeted sequentially (≤3). Our results show that phase maps derived from intracardiac electrograms can be a powerful tool to map atrial activation patterns, yet they can also be misleading due to inaccurate localization of the rotor tip depending on electrode resolution and distance to the wall. This should be considered to avoid ablating regions that are in fact free of rotor sources of AF. In our experience, ablation of rotor sites was not successful to stop fibrillation. Our comprehensive simulation framework provides the means to holistically benchmark ablation strategies *in silico* under consideration of all steps involved in electrogram-based therapy and, in future, could be used to study more heterogeneously remodeled disease states as well.

## Introduction

Atrial fibrillation (AF) is one of the major health challenges that modern societies are facing. AF is projected to develop in 25% of currently 40-year-old adults in their later life (Schnabel et al., 2015). Catheter ablation of the pulmonary veins (PVs) is the cornerstone of curative AF therapy and effective in 75% of patients with paroxysmal AF (Kuck et al., 2016). However, isolation of the PVs alone for persistent/permanent AF or AF with concurrent cardiac diseases is not sufficient in about half of the patients (Verma et al., 2015). Regrettably, several other ablation approaches did not yield better results in persistent AF patients in the large STAR AF II trial (Verma et al., 2015). Hence, the optimal ablation strategy for persistent AF patients remains an open question.

Lately, it has been suggested that AF may be driven by discrete reentrant drivers (Narayan et al., 2014; Haissaguerre et al., 2016). Ablation of rotational centers, so-called rotors, guided by electrograms acquired with basket catheters has attracted attention. However, the reported success rates are equivocal ranging from 21 to 82% (Narayan et al., 2014; Buch et al., 2016; Dukkipati and Reddy, 2017; Miller et al., 2017; Mohanty et al., 2018) demanding mechanistic investigation of this tailored ablation approach (Loewe and Dössel, 2017; Trayanova et al., 2018).

We present a framework to computationally evaluate basket catheter guided ablation mechanistically under controlled conditions. We tested this approach using an anatomically personalized model of a human AF patient. This study builds on our previous work (Alessandrini et al., 2017), which is extended in several ways: the sensitivity of the rotor trajectory estimated from catheter signals to the distance between the catheter and the atrial wall is studied; simulated acquisitions of a realistic Constellation basket catheter are considered in addition to simple 2D grid catheters; the basket-guided FIRM ablation protocol is simulated including the treatment by implementing up to three successive ablations.

## Materials and methods

### Clinical data

Magnetic resonance (MR) was used to image the atria of one paroxysmal AF patient selected for radio-frequency ablation. A 1.5 T MR scanner (Achieva, Philips Medical System) was used with a 3D spoiled gradient recalled sequence. Contrast enhanced 3D MR angiographic (MRA) images were acquired (echo time: 1.12 ms, repetition time: 3.74 ms, flip angle: 25°, in-plane resolution 0.7 × 0.7 mm^2^ and slice thickness 3 mm with 1.5 mm overlap) with gadolinium injection of 0.1 mmol/kg followed by a 20 ml saline flush. Acquisition was ECG triggered and in breath hold. The study was approved by the Ethics IRST, IRCCS AVR Committee (CEIIAV n. 1456 prot. 6076/2015 I.5/220). Informed consent was obtained from the subject.

### Anatomical model

**Figure 1:**
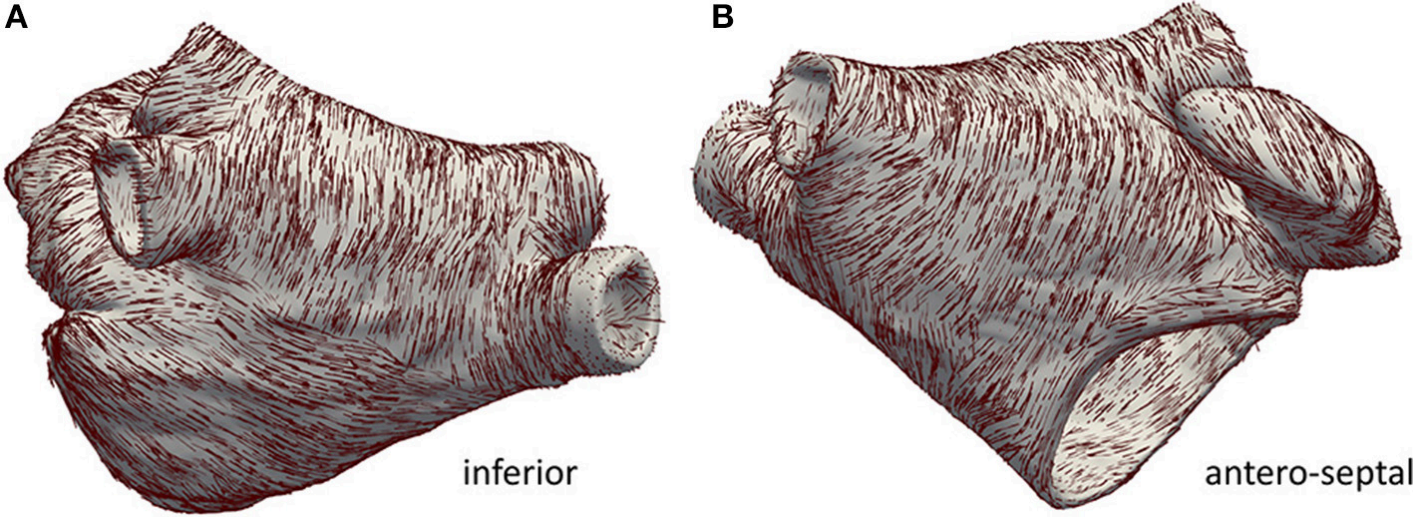
Anatomical model with annotated fiber bundles. In **(A)** the inferior wall is shown, in **(B)** the anterior/septal wall is shown.

**Figure 2:**
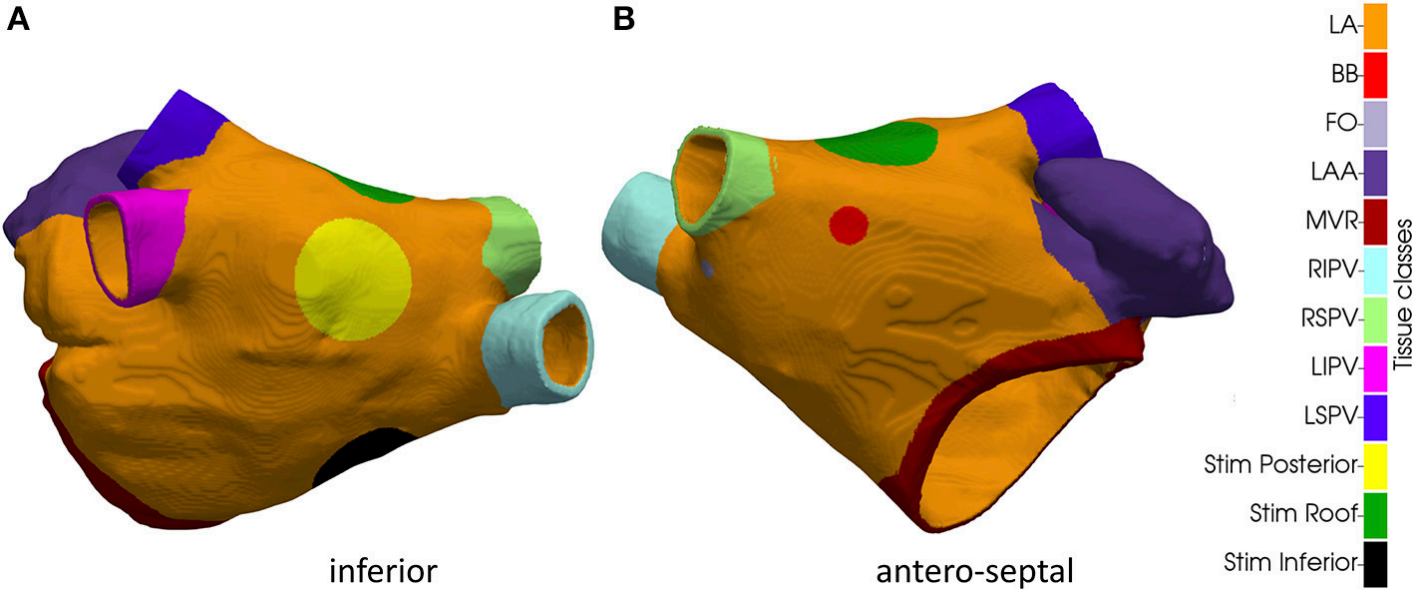
Inferior **(A)** and antero-septal **(B)** aspect of the anatomical model with tissue classes. LA bulk, Bachmann's bundle insertion point (BB), fossa ovalis (FO), left atrial appendage (LAA), mitral valve ring (MVR), right inferior pulmonary vein (RIPV), right superior pulmonary vein (RSPV), left inferior pulmonary vein (LIPV) left superior pulmonary vein (LSPV). The three circular regions in which the extra stimuli were applied to initiate reentry in the three simulated AF scenarios are indicated as well.

Left atrial (LA) blood pool, LA appendage (LAA) and the four pulmonary veins (PVs) were segmented semi-automatically from the MRA data using in-house software (Valinoti et al., 2018). The 3D segmentation was obtained by stacking 2D segmentations along the cross-slice direction. The segmented volume was resampled at a uniform voxel size of 0.33 × 0.33 × 0.33 mm^3^. Gaussian smoothing was employed to remove the staircase artifacts coming from the anisotropic voxel size of the MR system. The atrial epicardium was obtained by extruding the endocardial segmentation to a uniform wall thickness of 3 mm (Platonov et al., 2008; Krueger et al., 2013b). The LA model was augmented with myofiber directions in each cell of the anatomical model using a rule-based algorithm (Wachter et al., 2015). Hereto, a set of 13 anatomical landmarks was manually identified on the LA surface and used to identify the main fiber bundles. The resulting fiber distribution is illustrated in Figure 1. The same algorithm was used to label relevant tissue classes: atrial body, PVs, LAA and mitral valve ring (Krueger et al., 2013a). Additionally, Bachmann bundle and fossa ovalis were annotated manually and used to simulate sinus rhythm activation, as discussed in the following. Tissue classes are illustrated in Figure 2. Ablation lesions were modeled as non-conducting transmural structures.

### Simulating atrial activation

The monodomain model was used to simulate excitation propagation in the LA based on a heterogeneous version (Loewe et al., 2015) of the Courtemanche membrane model (Courtemanche et al., 1998) accounting for AF related ionic remodeling (Loewe et al., 2014). The monodomain conductivity and its anisotropy were set as in (Loewe et al., 2015) besides altering the common LA properties homogeneously to account for AF-induced remodeling (reduced conductivity 8.9 mS/m and increased anisotropy of 10). Sinus activation was simulated by pacing the model at the insertion point of the Bachmann bundle and at the Fossa Ovalis with a physiological delay of 26 ms (Loewe et al., 2016). In order to initiate reentry, an extra stimulus was placed in a circular area with a radius of 5 mm. By changing the location of this area it was possible, in several cases, to cause unidirectional block and subsequent reentry (see Results). Transmembrane voltages and current densities were computed by the monodomain reaction-diffusion solver acCELLerate (Seemann et al., 2010; Niederer et al., 2011) using a finite element scheme. The ordinary differential equations of the cell model were integrated using the Rush-Larsen scheme for the gating variables and a forward Euler scheme for the remaining variables. Constant time stepping of 20 μs was used. The membrane models were initialized in a single cell environment to reach steady-state at a basic cycle length of 800 ms.

### Multielectrode catheters

#### Grid catheters

**Figure 3:**
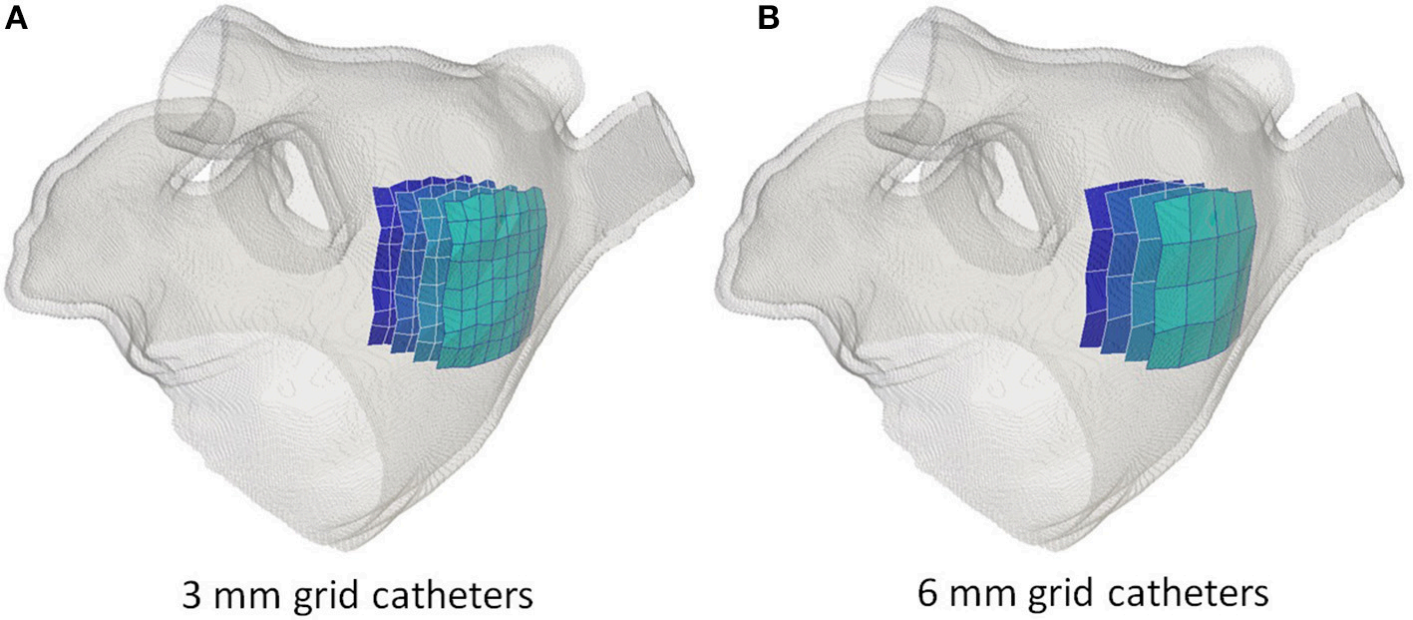
Simulated grid catheters. In **(A)** the catheter with a 3 mm inter-electrode spacing. In **(B)** the catheter with a 6 mm inter-electrode spacing. In both cases, four catheters are illustrated corresponding to a distance to the atrial wall of 0 mm (lightest blue), 5, 10, and 15 mm (darkest blue).

To study the sensitivity of rotor tracking to spatial sampling density (i.e., intra-electrode distance) and distance to the atrial wall, we employed two “toy” catheters. The two catheters were obtained by positioning 9 × 9 (respectively, 5 × 5) electrodes on a square grid with an inter-electrode distance of 3 mm (respectively, 6 mm), as shown in Figure 3. As such, the total size of the 2D patch was 24 × 24 mm^2^ in both setups. The 2D grid catheters were then positioned in the 3D atrial model such that the tissue region of interest was covered at a given distance from the atrial wall. Catheter positioning was implemented as follows. An initial 3D rigid transform was employed to align the grid catheter with a given region of interest. Hereto, the center point of the desired region of interest was selected manually on the atrial endocardium and used as origin of the local coordinate system. The set of endocardial mesh nodes falling within a radius of 5 mm from the selected point were then used to fit a 2D plane and, hence, define the 3 axes of the local coordinate system: two tangential to the endocardial surface and one perpendicular to it. After 3D rotation, the catheter was adapted to the specific atrial shape by repositioning each electrode to its closest position on the atrial endocardium. To modify the distance between the electrode and the atrial wall, all electrodes were moved rigidly along the inwards normal direction defined by the local coordinate system used for rotation.

#### Basket catheters

**Figure 4:**
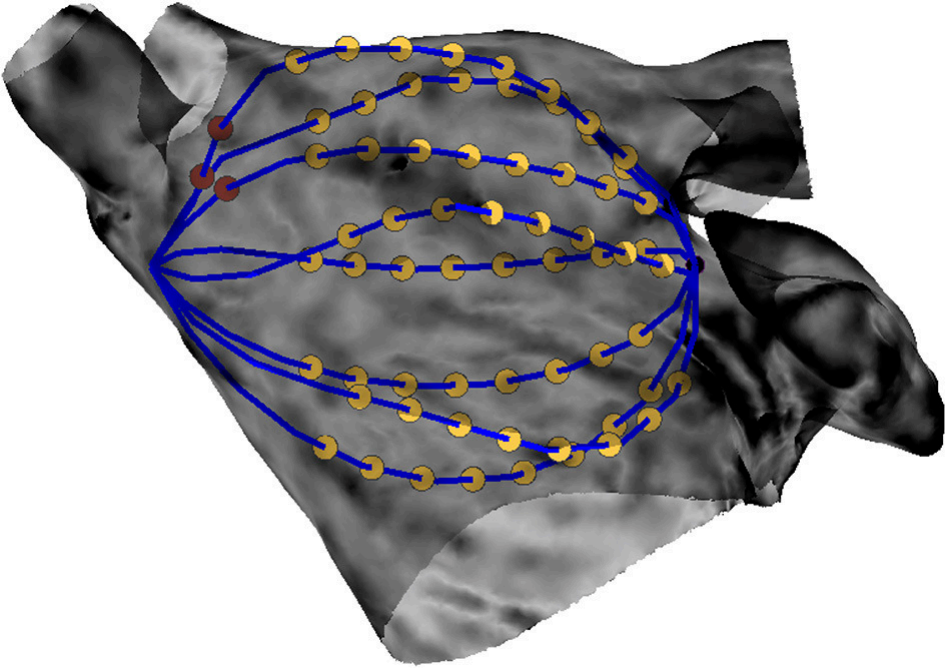
Simulated basket catheter within the LA geometry. Yellow dots denote the 64 electrodes of the catheter. Blue lines illustrate the catheter's splines. One and two red dots indicate the proximal end of spline A and spline B, respectively.

We used the algorithm presented in Oesterlein et al. (2016) to position a simulated basket catheter inside the specific atrial anatomy. In brief, a set of Frenet-Serret parameterized spline pairs of a given size are deformed to minimize the inner tension energy while fulfilling the constraints posed by the atrial surface as well as the distal and proximal end of the basket catheter. We chose the Constellation basket size that best fit our geometry. Hereto, a commercially available 48 mm basket with 8 splines and 4 mm inter-electrode spacing was positioned inside the atrial geometry. The position was optimized with the objective of maximizing the coverage of the rotor trajectory with basket electrodes. The basket catheter is illustrated in Figure 4.

### Simulated electrograms

Unipolar electrograms (EGMs) ϕ(**x**, *t*) at point electrode positions x were computed from the monodomain current density distributions *I*(**x**, *t*) under the simplifying assumption of an unbounded volume conductor:

$$\begin{array}{l} \phi \left(\text{x},t\right)=\frac{1}{4\pi \sigma }\underset{V}{\sum }\frac{I\left({\text{x}}_{i},t\right)}{\left||{\text{x}}_{i}-\text{x}|\right|} \end{array}$$

where σ is the electrical conductivity of the volume conductor, x is the sampling position (i.e., the electrode position), x_i_ is the coordinate of the ith cell of the atrial mesh and V comprises the whole atrial wall. Simulated EGMs were sampled at 1 kHz.

### Rotor tracking from multi-electrode acquisitions

We used the method presented in Valinoti et al. (2017) to detect and track electrical reentries from the simulated electrograms. Briefly, rotor detection is based on the concept of phase singularities (PS). Hereto, the simulated electrograms were first converted into phase signals by using a modified version of the sinusoidal recomposition approach (Kuklik et al., 2015). The extracted phase values where then mapped from the electrode positions to the 3D geometry by nearest neighbor interpolation with a cut-off value of 13 mm. The algorithm then looked for closed loops with monotonically increasing/decreasing phase values and a jump of at least π as per definition of a PS. The search was progressively extended to neighboring regions, i.e., sharing an interface in the 3D reconstruction. Rotors were identified by tracking the persistence of PS in the same region in subsequent time frames. The PS persistence was estimated considering a threshold on the maximum distance between the coordinates of its location estimated frame by frame. The threshold was fixed at 8 mm for the 3 mm grid, 17 mm for the 6 mm grid and 20 mm for the basket. As such, a tip trajectory was estimated for each rotor in each of the simulations and the rotor with the longest lifespan was considered as ablation target.

### Trajectory density maps and evaluation of tracking accuracy

The ground truth rotor tip trajectory was tracked from the high resolution electrical activation maps. Hereto, the transmembrane potential time courses V_m_(t, **x**_i_) of each vertex **x**_i_ were converted into phase signals by computing the phase of the complex-valued analytical signal z(t, **x**_i_):

$$\begin{array}{l} z\left(t,\;x_{i}\right)=V_{m}\left(t,\;x_{i}\right)+jH\left\{V_{m}\left(t,\;x_{i}\right)\right\}, \end{array}$$

with H{} being the Hilbert transform and j being the imaginary unit. The rotor tip was identified as a singularity in the reconstructed phase maps. Tracking accuracy was then assessed by comparing the trajectory estimated by the tracking algorithm vs. the ground truth. Hereto, instead of a pointwise comparison of the two trajectories, we used “trajectory density maps” as a way to represent temporal persistence of a rotor in a given atrial region over a given time interval Δ*t*. If we denote by **y**(*t*_*j*_) the rotor tip position at time *t*_*j*_, then the density value d_i_ at a mesh node **x**_*i*_ was computed as:

$$\begin{array}{l} d_{i}=A\cdot \underset{t_{j}\in \Delta t}{\sum }f\left(\left||\text{y}\left(t_{j}\right)-{\text{x}}_{i}|\right|\right),\text{ with }f\left(z\right)=e^{-z^{2}/2\sigma^{2}} \end{array}$$

where σ defines the spatial extent of the smoothing kernel *f*. *A* is used to normalize peak density to one. In this study, we used an integrating time interval Δ*t* = 2 s and σ = 3 mm. Hence, tracking accuracy was evaluated by comparing the position of the peak in the estimated trajectory map **d** vs. the ground truth one as ultimately, peak density was used to guide rotor ablation, as described in the following.

### Simulated rotor-ablation protocol

Radiofrequency ablation was simulated by setting zero conductivities (longitudinal and transverse) for mesh elements pertaining to the ablation lesions (circular regions of 7 mm radius Bayer et al., 2016, unless otherwise specified). For a given tissue simulation and positioning of the multi-electrode catheter, rotor-driven ablation was simulated by repeating the following steps:

- EGM signal recordings were simulated for a period of 2 s, as described in section Simulated electrograms;
- The rotor trajectory was reconstructed from the simulated EGM signals with the custom rotor tracking algorithm, as described in section Rotor tracking from multi-electrode acquisitions;
- The trajectory density map was computed from the estimated rotor trajectory, as described in section Trajectory density maps and evaluation of tracking accuracy;
- The circular ablation site was centered in the maximum of the estimated density map;

As such, up to three ablations were incrementally applied.

## Results

### Simulation of rotor-based sustained atrial activation

Using the methods described in section Simulating atrial activation it was possible to induce complex propagation patterns maintained by stable rotors. In particular, we obtained three different sustained patterns by changing the location of the extra stimulus applied to induce reentry: one at the inferior wall (“Simulation Inferior,” Supplemental Video 1); one at the posterior wall toward the roof (“Simulation Posterior,” Supplemental Video 2); one at the roof (“Simulation Roof,” Supplemental Video 3). The position of the extra stimulus used to induce reentry in the three cases is illustrated in Figure 2.

**Figure 5:**
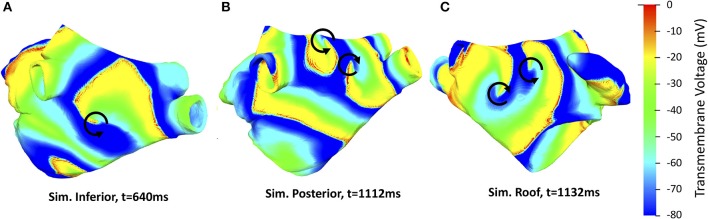
Representative screenshots of the three AF simulations considered with reentries indicated by black arrows. Color encodes the transmembrane voltage V_m_. A counter-clockwise rotor is located on the low posterior wall in Simulation Inferior **(A)**, a figure of eight pattern is present on the roof of Simulation Posterior **(B)** and a counter-clockwise reentry on the high anterior wall and a clockwise reentry below the right superior PV is present in Simulation Roof **(C)**.

In all cases, this procedure generated “figure of eight” reentries (i.e., two reentries with opposite chirality). In Simulation Inferior the counter-clockwise rotor was located in the low posterior wall moving toward the septum below the right inferior PV in the last part of the simulation. The clockwise rotor was stably located at the septum, above the mitral valve. In Simulation Posterior a rather stable figure of eight was formed by two highly interacting opposite reentries, which were located close to each other: one on the high posterior wall roughly midway between the right and left inferior PVs and the second one on the roof toward the left superior PV. At *t* = 1.05 s, a second figure of eight appeared at the septum, below the right superior PV. In Simulation Roof, one counter-clockwise reentry was stable in the high part of the anterior wall while a clockwise reentry was initially centered at the roof toward the right PVs. At time *t* = 0.8 s, a third, clockwise, reentry started below the right superior PV, most likely due to wavebreak caused by the local change in fiber direction. Consequently, the second reentry gradually shifted toward the posterior wall and vanished, while a stable figure of eight was formed by the first and third reentries. Representative screenshots are shown in Figure 5 while the videos are provided in the Supplemental Material.

### Simulation of grid catheters: effect of electrode-wall distance and inter-electrode distance on rotor tracking

**Figure 6:**
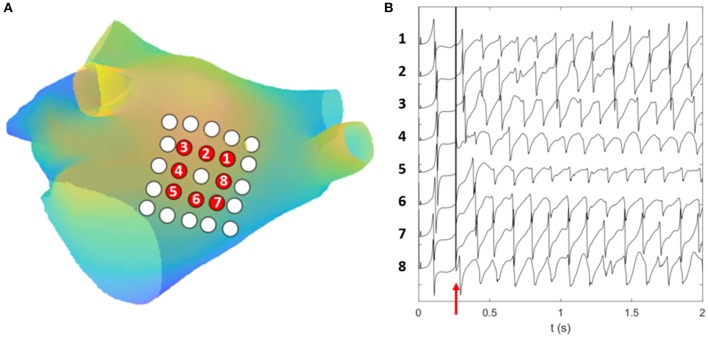
Simulated EGM signals. **(A)** Computational geometry with the 25 electrodes of the 6 mm grid catheter. Electrodes for which the EGM signals are displayed are colored in red. They are numbered in counter-clockwise order following the direction of rotation of the simulated rotor. **(B)** The simulated EGM signals computed for the electrodes colored in red on the first 2 s of Simulation Inferior. The y axis of **(B)** reports the electrode number. The red arrow in **(B)** indicates the time at which the extra stimulus was applied to induce reentry.

For each of the three simulated excitation patterns, we computed the electrograms and the corresponding phase maps from eight grid catheters, characterized by different inter-electrode spacing (3/6 mm) and distances to the atrial wall [0 mm (i.e., in contact), 5, 10, and 15 mm]. Example of simulated EGM signals for the 6 mm catheter from Simulation Inferior are illustrated in Figure 6. The grid catheters were positioned to cover as much as possible of the ground truth rotor trajectory and were used to assess the rotor tracking algorithm's sensitivity to electrode spacing and wall distance.

**Figure 7:**
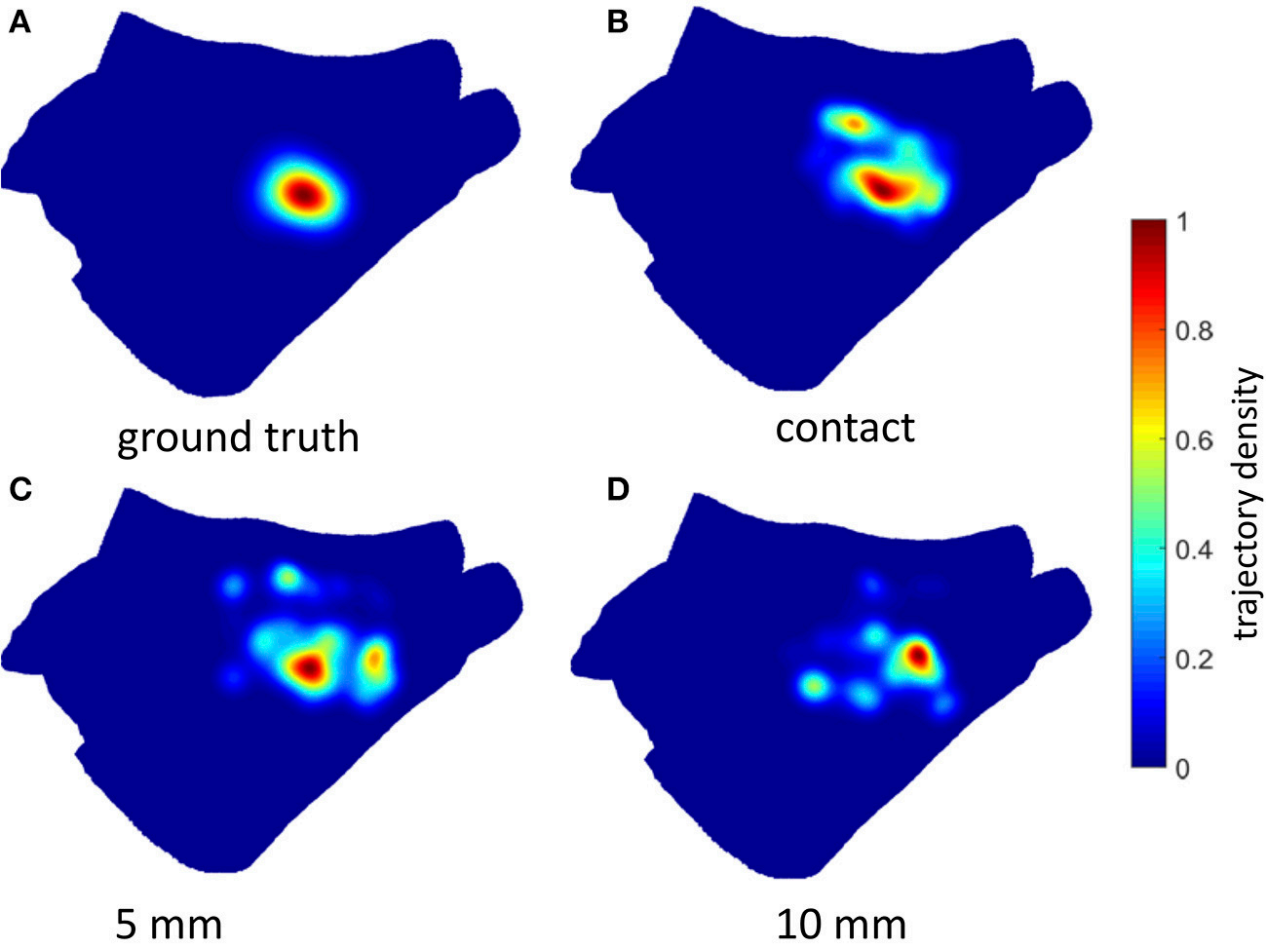
Density maps of rotor tip trajectory reconstructed by the 3 mm grid catheter at different distances from the atrial wall in Simulation Inferior. **(A)** Ground truth density map; **(B)** 3 mm grid catheter in contact; **(C)** 3 mm grid catheter at 5 mm from the wall; **(D)** 3 mm grid catheter at 10 mm from the wall. Color encodes the trajectory density value.

**Figure 8:**
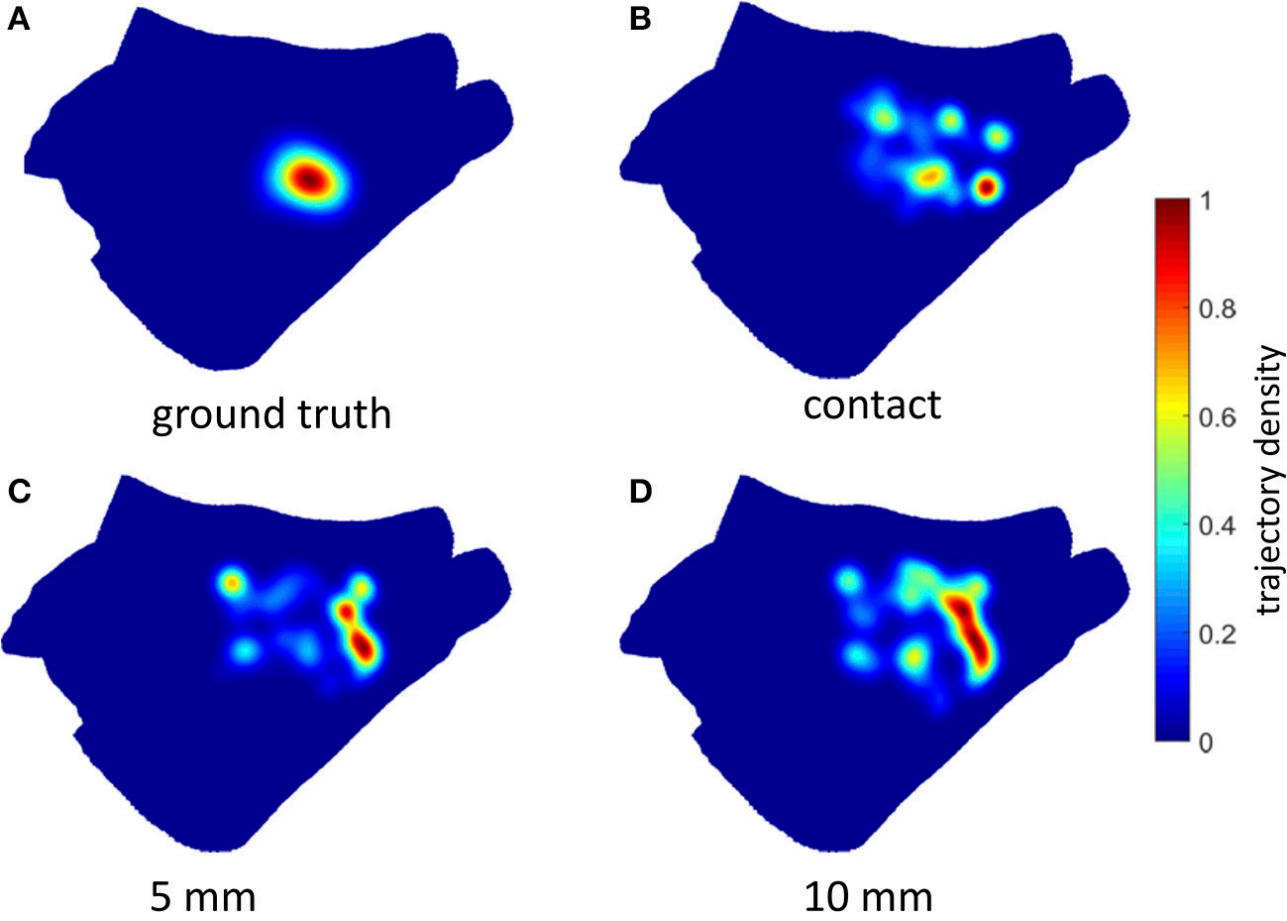
Density maps of rotor tip trajectory reconstructed by the 6 mm grid catheter at different distances from the atrial wall in Simulation Inferior. **(A)** Ground truth density map; **(B)** 6 mm grid catheter in contact; **(C)** 6 mm grid catheter at 5 mm from the wall; **(D)** 6 mm grid catheter at 10 mm from the wall. Color encodes the trajectory density value.

The estimated rotor tip density maps in the case of Simulation Inferior are shown in Figure 7 (3 mm inter-electrode spacing) and Figure 8 (6 mm inter-electrode spacing). Due to the reduction of the spatial resolution of the electrical activity by sampling with a limited number of electrodes, several spurious peaks in the rotor tip density maps were observed. Nevertheless, when the distance from the atrial wall was below 10 mm, it was possible to correctly locate the maximum peak density (error < 9 mm) using the 3 mm inter-electrode spacing catheter (Figure 7).

On the contrary, when using 6 mm inter-electrode spacing, a systematic error (>11 mm) in the location of the density peak was observed independently of the distance from the atrial wall (Figure 8). The accuracy of the rotor tracking algorithm on all three simulated activation patterns is presented in Table 1.

**Table 1:** Peak to peak distance (in mm) between ground truth and estimated rotor density map from grid catheter with different resolution (3 or 6 mm inter-electrode distance) and distance from the atrial wall.

		**Distance from the atrial wall**			

		**0 mm (contact)**	**5 mm**	**10 mm**	**15 mm**
Grid resolution	3 mm	1.2 (0.9/1.7/1.9)	5 (0.6/3.9/ 0.5)	8.4 (8.9/3.9/12.4)	11.5 (11.7/9.0/13.8)
	6 mm	11.8 (12.9/15.8/6.6)	10.3 (12.6/6.0/12.3)	12.1 (11.7/6.5/18.2)	16.4 (16.0/18.5/14.6)

*Distances are given as average value and individual values for all three excitation patterns (Simulation Inferior/Simulation Posterior/Simulation Roof), for each distance-resolution combination*.

### Simulation of sequential ablation of rotors based on basket catheter mapping

For each simulation, the acquisition from a realistic basket geometry (cf. section Basket catheters) was analyzed to locate rotors as potential targets for ablation.

#### Simulation inferior

**Figure 9:**
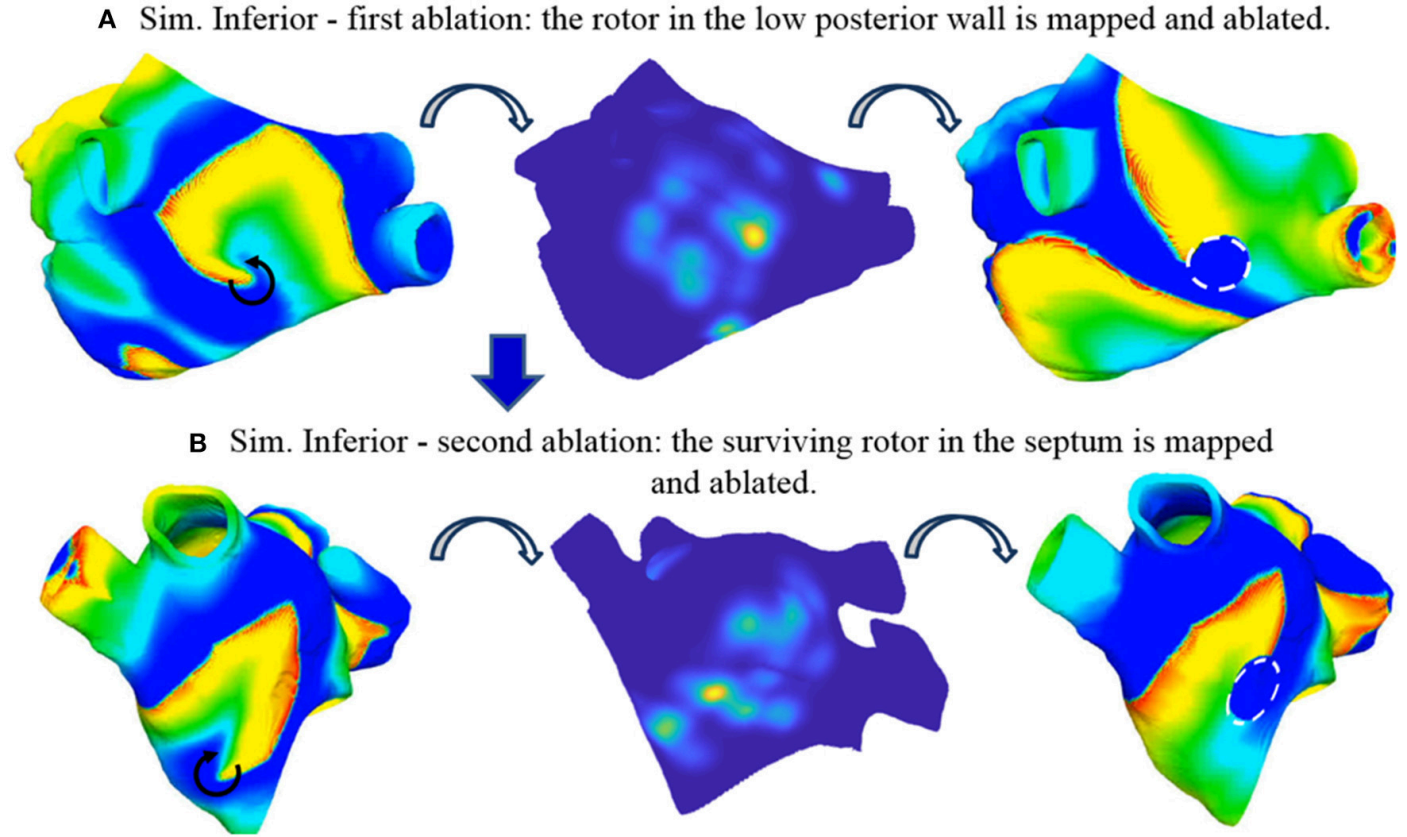
Simulated ablation of Simulation Inferior. Two reentries are present before ablation: one in the posterior wall (**A**, left) and one in the septum (not visible from this view, see Supplemental Video 4). The basket-reconstructed density map identifies the first reentry (**A**, mid) which is ablated with a circular region (**A**, right, dashed white circle). After ablation, AF is sustained by the second reentry in the septum (**B**, left), which is mapped **(B**, mid) and ablated (**B**, right, dashed white circle). Two coupled reentries arose around the anatomical obstacles formed by the two lesions induced by sequential ablation (see Supplemental Videos 4–11). Color code is identical to Figure 5 (left and right) and Figure 7 (mid).

The sustained activity (Supplemental Videos 1, 4) was mapped with the basket catheter (Figure 4) and the corresponding rotor tip density map (Figure 9A, mid) correctly located a peak at the low posterior wall, where one of the driving rotors was meandering. While only a smaller amplitude peak in the density map was found in correspondence of the septal rotor. The highest peak was targeted with circular tissue ablation (Figure 9A, mid), which caused a reentry around the lesion first, which was then (at *t* = 2.5 s) overdriven by the second rotor located in the septum above the mitral valve, which was unaffected by this ablation (Supplemental Video 5). An illustrative screenshot showing the stable rotor on the septum is depicted in Figure 9B (left).

We then tested the effect of ablation lesion size. By reducing the dimension of the circular lesion, it was possible to change its interaction with the reentry, however without stopping it. A 3.5 mm radius lesion was the smallest one which caused reentry around the lesion (Supplemental Video 6). A 2 mm radius lesion interacted with the spiral but was too small to sustain the reentry around it; the reentry extended inferior-septally and toward the roof to form a larger functional obstacle first. Later on, the core moved toward the roof and the lesion did not play a role anymore (Supplemental Video 7). A 1 mm radius lesion did not affect the reentry at all (Supplemental Video 8).

The stable rotor in the septum, which became dominant after the first ablation (Supplemental Video 5) was then mapped again with the 8-spline basket (Figure 4). In this case, the localization of the rotor tip based on the density map was less precise (Figure 9B, mid), probably due to the low-resolution basket coverage of the rotor. The peak of the density map was targeted by a second circular ablation (Figure 9B, right). This second ablation (Supplemental Video 9) led back to a pattern similar to the initial one: two coupled (figure of eight) reentries arose around the anatomical obstacles formed by the two lesions induced by sequential ablation.

Linear lesions toward anatomical obstacles were tested in order to stop reentry but were unsuccessful. A linear ablation connecting the lesion at the septum with the mitral valve was able to stop the reentry anchored around that lesion but did not affect the reentry around the other lesion, therefore it was not sufficient to stop sustained activity (Supplemental Video 10). An additional linear ablation connecting the lesion at the low posterior wall with the right inferior PV induced a combined larger reentry around the two lesions and the anatomical obstacles (Supplemental Video 11).

#### Simulation posterior

**Figure 10:**
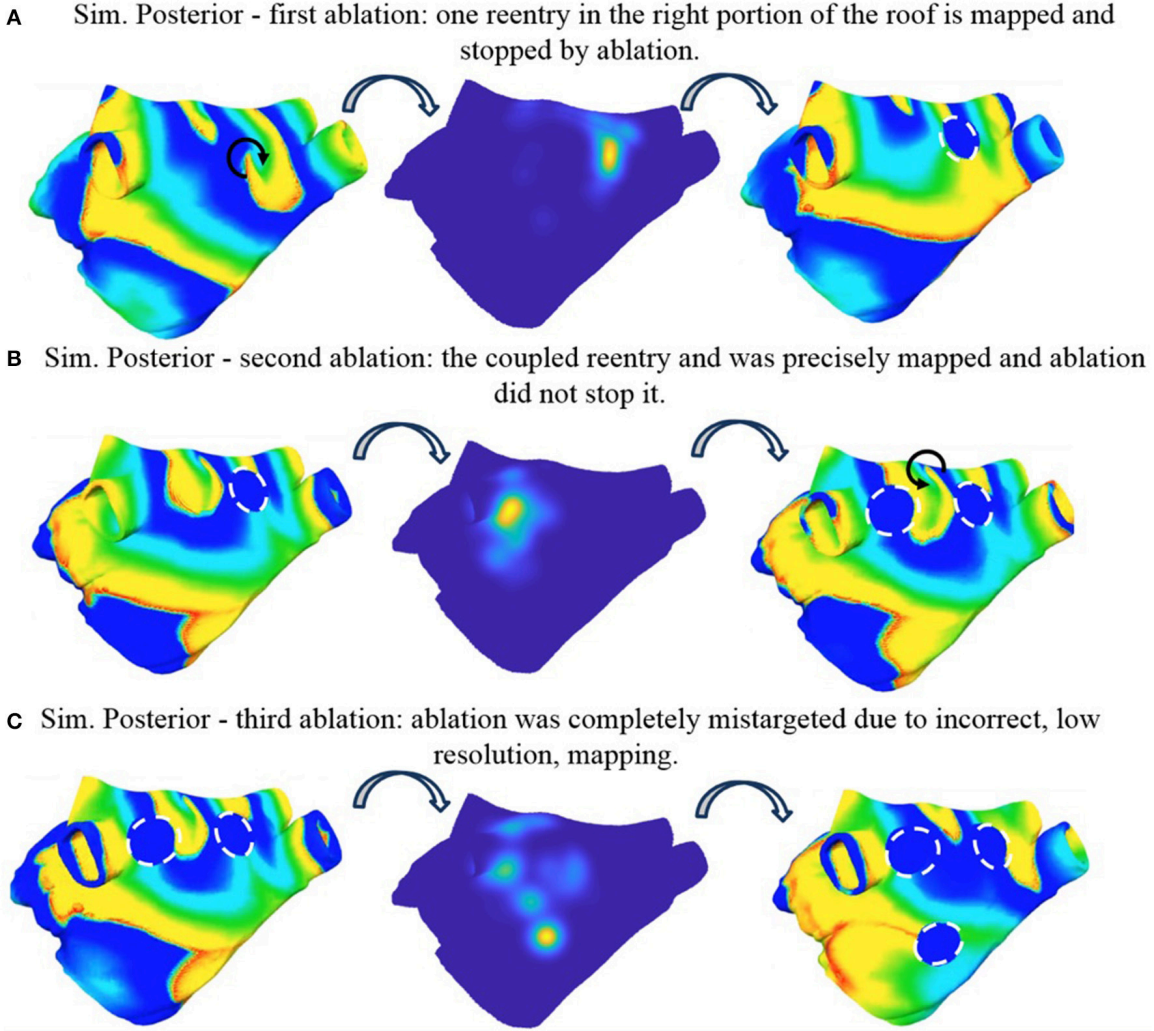
Simulated ablation of Simulation Posterior. Three ablations are applied incrementally. First ablation correctly identifies one reentry in the right portion of the roof and stopped it **(A)**. The second ablation was not precisely targeted to the coupled reentry and was not able to stop it **(B)**. The third ablation was completely mistargeted due to incorrect, low resolution mapping **(C)** (see Supplemental Videos 2, 12–14). Color code is identical to Figure 5 (left and right) and Figure 7 (mid).

The sustained activity shown in Supplemental Video 2 was mapped with the basket catheter (Figure 4); the corresponding rotor tip density map (Figure 10A, mid) correctly pinpoints a peak at the roof, where one of the driving rotors was meandering. This peak was targeted by a circular ablation (Figure 10A, right), which stopped the reentry without affecting neither the coupled reentry nor the other figure of eight located at the septum (Supplemental Video 12). Illustrative screenshots after the ablation are shown in Figure 10A (right) and Figure 10B (left). The atrium was mapped again with the 8-spline basket (Figure 4), in the attempt to locate the second rotor at the roof as a target for ablation. But it was not possible, based on the density map, to precisely locate the rotor tip. Indeed, the peak was clearly shifted to the left, toward the left PVs, with respect to the actual rotor tip (Figure 10B, mid). By trusting the results of the low-resolution catheter map, i.e., ablating a circular region centered in the position of the density peak, it was not possible to stop the second rotor, which indeed kept rotating close to the rather narrow space between the two lesions (Figure 10B, left, and Supplemental Video 13). However, after the second ablation, the rotor moved slightly toward the anterior wall leading to a collision with one of the two rotors at the septum. At the end, only two coupled rotors survived. When a third rotor-guided ablation was performed as intended in the FIRM protocol (Figure 10C and Supplemental Video 14), the sustained activity did not stop. The rotors moved slightly and after a transient phase, two reentries became stable: a rotor in the anterior wall and an anatomical reentry around the right superior PV.

#### Simulation roof

**Figure 11:**
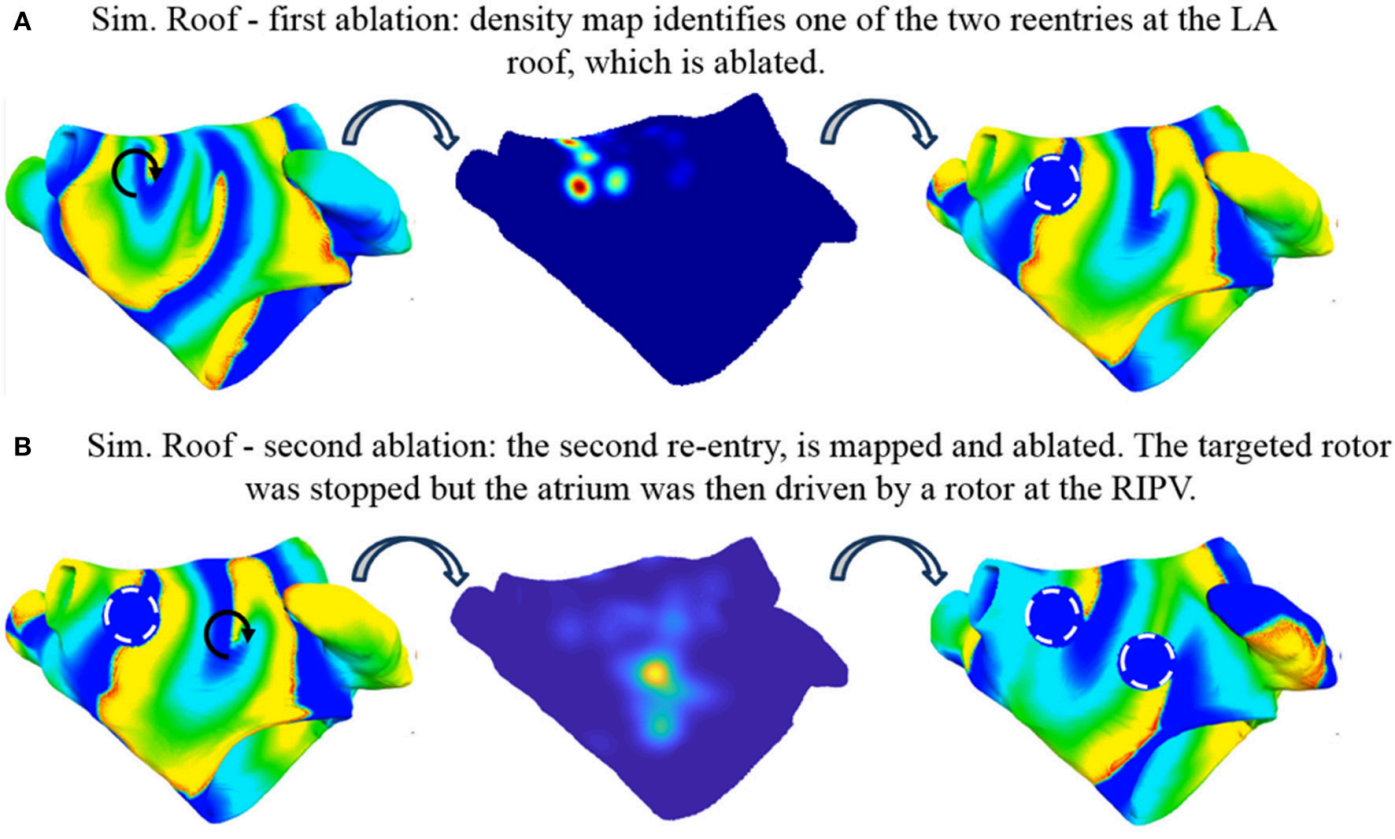
Simulated ablation of Simulation Roof. Two coupled reentries are present before ablation (**A**, left). The basket-reconstructed density map identifies one of them (**A**, mid) which is ablated with a circular region (**A**, right, dashed white circle). After ablation, AF is sustained by the second reentry (**B**, left), which is mapped (**B**, mid) and ablated (**B**, right, dashed white circle). The targeted rotor was stopped but the atrium was then driven by a rotor at the right inferior PV (see Supplemental Videos 3, 15, 16). Color code is identical to Figure 5 (left and right) and Figure 7 (middle).

The sustained activity shown in Supplemental Video 3 was mapped with the basket catheter (Figure 4) and the corresponding rotor tip density map (Figure 11A, Mid) correctly pinpoints a peak below the right superior PV, where one of the driving rotor was meandering. This peak was targeted by a circular ablation, which stopped this reentry. One rotor remained at the anterior wall while a second one appeared at the right inferior PV (Supplemental Video 15). An illustrative screenshot after the ablation is shown in Figure 11A (Left).

The atrium was mapped again with the basket (Figure 4) to locate the rotor at the anterior wall as a target for ablation. After the second ablation, the targeted rotor was stopped (Figure 11B); after an initial reentry around the anatomical obstacles produced by the ablations the disorganized activity in the entire atrium was driven by the rotor at the right inferior PV (Supplemental Video 16). Due to its position, within the initial tract of the PV, it was not possible to map this rotor with the basket catheter.

## Discussion

### Main findings

The accuracy of localizing rotors using multielectrode catheters and the efficacy of ablating the derived targets cannot be easily assessed *in vivo*, since the actual fibrillatory activation patterns are not known in great detail in clinical practice. We have implemented a computational framework to benchmark basket catheter guided ablation *in silico*.

Our results show that (i) in a simulated homogeneously remodeled atrium, stable rotors can be induced in different regions, depending on the timing and the location of an extra stimulus; this means that rotors are not constrained to unique anatomical structures or locations; (ii) Rotors may be identified and located with clinically sufficient accuracy by phase maps built from basket catheter recordings only if resolution and distance from the wall are appropriate; (iii) Ablation of rotor sites does not stop reentry in homogeneously remodeled atria.

### Simulation of rotor-based sustained atrial activation

It was possible to induce rotor-based sustained atrial activity by using a model that does not take into account transmural heterogeneity/uncoupling nor the presence of fibrosis. Not considering these effects is of course a limitation of the model and further studies with more detailed models are warranted. On the other hand, our results demonstrate that such elements are not strictly needed in order to obtain stable rotors in human atria and that stable rotors are not unique patterns constrained by the presence of a specific heterogeneous substrate. Zhao et al. (2017) used a 3D human heart-specific atrial model integrating local wall thickness and transmural fibrosis data and found that reentrant AF drivers were uninducible when fibrosis and myofiber anisotropy were removed from the model. Taken together these results suggest that myofiber anisotropy, which was included in our model too, can be the key factor for inducibility. On the other hand, also the patient-specific anatomy or the different pacing protocols used to induce the reentrant activity might play a role. In particular, in a preliminary study we were not able to induce reentry by using a single point burst pacing protocol.

In our hands, while keeping exactly the same substrate, stable rotors were induced in multiple different positions of the left atrium. Thus, it is not surprising that ablation of the rotor core might be unsuccessful since rotors can move or arise somewhere else in the atrium. In many computational studies, this aspect was not investigated since only one sustained activation pattern was analyzed (e.g., Martinez-Mateu et al., 2018) or different patterns were induced by changing the underlying substrate (e.g., Vigmond et al., 2004 by changing the acetylcholine level). However, in some previous studies (McDowell et al., 2012; Gonzales et al., 2014; Krueger et al., 2014; Zhao et al., 2017) rotors were shown to be sustained and stabilized by fibrosis, and more recently a relationship between fibrosis and reentrant activity has been clinically reported (Cochet et al., 2018). Nevertheless, the causal effect and the impact of fibrosis on the ablation success rate is not completely clarified yet and deserves further studies. With this study, we contribute insight based on a simple structural model with a limited number of free parameters, which can be well controlled and analyzed.

### Simulation of electrogram acquisition: effect of electrode-wall distance and inter-electrode distance on rotor tracking

Rotors in our simulations were not perfectly stable in space, some meandering was always observed. However, by tracking their movement, a quite concentrated ground truth core trajectory density was observed (Figures 7, 8, Top Left). Conversely, when looking at the estimated core trajectory density based on simulated electrogram recordings, a number of spurious peaks were always present (Figures 8, 9) even for catheters in contact with or close to the endocardial wall. This is mainly due to the discretization, i.e., the low resolution of the EGM acquisition as shown by Roney et al. (2017). Different kinds of interpolation were suggested to increase the apparent spatial EGM sampling resolution and therefore the resolution of the estimated phase map. We preferred not to interpolate for two reasons: (i) It has recently been shown in a simulation setting quite similar to ours that interpolation between electrodes can generate artifact “phantom” rotors (Martinez-Mateu et al., 2018). (ii) We wanted to reproduce phase maps similar to those clinically available in the Topera format with the relevant difference that maps are directly computed on a patient-specific atrial anatomy rather than a fixed isotropic 8 × 8 grid (Oesterlein et al., 2016). Due to the observed dispersion of estimated core trajectories, it seems important to rely on an integral measure such as the density peak over time.

Localization of rotors worked well (i.e., with errors less or equal to the dimension of a lesion produced by the ablating catheter in a real procedure) for inter-electrode spacing of 3 mm and distances to the wall below 10 mm. This is in agreement with the recent observations by Martinez-Mateu et al. (2018) that rotor detection by the basket maps varied depending on the basket's position and the electrode-wall distance. While it is difficult to establish a cut-off threshold for these parameters, two warnings can be derived for clinical panoramic mapping with basket catheters: (i) it is important to include an estimate of the distance of each electrode from the wall when estimating the phase map in order to exclude those too far to give reliable contributions, (ii) the commercially available basket catheters seem not to assure a sufficient resolution if used in the fully open position to simultaneously acquire EGMs from (almost) the entire atrial chamber.

### Simulation of sequential ablation of rotors based on basket catheter mapping

We found that ablation of rotor sites did not stop reentry in homogeneously remodeled atria. This was observed for all three reentry scenarios and independent from lesion size. By computing the phase maps and then the rotor trajectories directly on the atrial anatomy, it was possible to define the targets for rotor ablation very precisely. This could prove to be better than analysing and visualizing the phase map in a two-dimensional surface and then manually locate the ablation target on the atrial anatomy (Oesterlein et al., 2016). Indeed, as shown in one of our simulated scenarios (Simulation Posterior), relatively modest errors in rotor localization might lead to ablation with no effect on the rotor itself. This underlines the importance of sufficient spatial resolution and coverage when using panoramic mapping to guide rotor ablation.

Several AF ablation strategies have been studied using computational models as reviewed by Zhao et al. (2015) and Jacquemet (2016). Besides rotor ablation, sites of local electrical dyssynchrony have recently been studied as potential targets for ablation *in silico* (Kuklik et al., 2016). Compared to previous *in silico* studies of rotor ablation (McDowell et al., 2012, 2015; Gonzales et al., 2014; Krueger et al., 2014; Ugarte et al., 2015; Bayer et al., 2016; Hwang et al., 2016; Zahid et al., 2016; Lim et al., 2017), the work presented here is, to the best of our knowledge, the first which considers the full workflow from signal acquisition using realistic catheter models via rotor tip estimation to virtual ablation.

While most previous studies considered spatial fibrosis distribution (McDowell et al., 2012, 2015; Gonzales et al., 2014; Krueger et al., 2014; Bayer et al., 2016; Zahid et al., 2016), we chose to focus on the basic mechanisms in a homogeneously remodeled substrate. Lim et al. (2017) chose a similar homogeneous approach, however high dominant frequency regions were targeted instead of rotors. Ugarte et al. (2015) only considered linear ablation patterns. Hwang et al. (2016) targeted rotors with virtual ablation in 2D and 3D models and obtained similar results. Sequential phase singularity-based ablation with a radius of 1 mm up to a maximum ablated area of 5% of the LA did not stop the arrhythmia. Instead, the rotor turned to a reentry encircling the ablated area. Combination of these findings is well in line with our results (observing sustained reentry with both permanent and transient anchoring to ablation lesions) as well as clinical data reporting that the majority of atrial tachycardias in patients undergoing a repeat procedure after prior FIRM guided ablation appeared to have little relationship to previous FIRM ablation sites (Latanich et al., 2018).

### Limitations

The model employed in this study has several limitations. While considering gross anatomy and well-established atrial electrophysiology and structure, heterogeneous fibrosis distribution and transmural uncoupling are relevant factors that were intentionally neglected in this study. Both these aspects are integral aspects of current hypotheses on AF mechanisms (Schotten et al., 2016) and can lead to spatially heterogeneous alterations of conductive and ionic properties. However, we chose to focus on studying the fundamental principles in a simple setting of homogeneous remodeling, first. Moreover, variability in the LA anatomy as well as the interplay between excitations in the left and right atrium are beyond the scope of this study. Moreover, the algorithm for phase analysis in (cf. section Rotor tracking from multi-electrode acquisitions) might decrease its accuracy in the case of fractionated electrograms, since sinusoidal recomposition is expected to result in a single phase inversion detection in correspondence of a local atrial activation characterized by two deflections or more. However, this evaluation falls out of the scope of this paper.

Despite these limitations, our work can be considered a promising starting point to build a clinically relevant computational framework to benchmark basket catheter guided ablation and allows, in its present form, to investigate and discuss basic relations.

## Conclusion

We presented a simulation framework covering the whole cycle from excitation propagation to electrogram acquisition and processing to virtual ablation. The application to basket catheter guided rotor ablation suggests that local ablation of rotor tips does not terminate reentry in homogeneously remodeled atria. While phase maps based on intracardiac catheter electrograms are a powerful tool to map atrial activation patterns, they can also mislead physicians due to inaccurate localization of the rotor tip depending on electrode resolution and distance to the wall. This might entail ablation in atrial regions that are in fact free of rotor sources of AF.

## Author contributions

MA conception of the study, software development, simulations, analysis of the results, manuscript drafting, figure preparation, revision of manuscript. MV image acquisition and processing, software development, analysis of simulated EGMs, phase mapping, rotor detection. LU software development, simulation of basket catheter, revision of the manuscript TO simulation of basket catheter, software development. OD supervision of the study. CC conception of the study, image processing, software development, analysis of the results, figure preparation, revision of manuscript. AL conception of the study, software development, simulations, analysis of the results, manuscript drafting, video preparation, revision of manuscript. SS conception of the study, software development, analysis of the results, manuscript drafting, revision of manuscript, supervision of the study.

### Conflict of interest statement

The authors declare that the research was conducted in the absence of any commercial or financial relationships that could be construed as a potential conflict of interest.
